# Multipotent vascular stem cells contribute to neurovascular regeneration of peripheral nerve

**DOI:** 10.1186/s13287-019-1317-7

**Published:** 2019-08-03

**Authors:** Ching-Wen Huang, Yuan-Yu Hsueh, Wen-Chin Huang, Shyam Patel, Song Li

**Affiliations:** 1 Department of Bioengineering, University of California, Berkeley, 94720 USA; 2 Division of Plastic and Reconstructive Surgery, Department of Surgery, National Cheng Kung University Hospital, College of Medicine, National Cheng Kung University, Tainan, 70456 Taiwan; 3 International Research Center for Wound Repair and Regeneration, National Cheng Kung University, Tainan, 70456 Taiwan; 4 Department of Bioengineering, University of California, CA, Los Angeles 90095 USA; 5 Department of Medicine, University of California, CA, Los Angeles 90095 USA

**Keywords:** Multipotent vascular stem cell, Stem cell therapy, Peripheral nerve regeneration, Blood-nerve barrier

## Abstract

**Background:**

Neurovascular unit restoration is crucial for nerve regeneration, especially in critical gaps of injured peripheral nerve. Multipotent vascular stem cells (MVSCs) harvested from an adult blood vessel are involved in vascular remodeling; however, the therapeutic benefit for nerve regeneration is not clear.

**Methods:**

MVSCs were isolated from rats expressing green fluorescence protein (GFP), expanded, mixed with Matrigel matrix, and loaded into the nerve conduits. A nerve autograft or a nerve conduit (with acellular matrigel or MVSCs in matrigel) was used to bridge a transected sciatic nerve (10-mm critical gap) in rats. The functional motor recovery and cell fate in the regenerated nerve were investigated to understand the therapeutic benefit.

**Results:**

MVSCs expressed markers such as Sox 17 and Sox10 and could differentiate into neural cells in vitro. One month following MVSC transplantation, the compound muscle action potential (CMAP) significantly increased as compared to the acellular group. MVSCs facilitated the recruitment of Schwann cell to regenerated axons. The transplanted cells, traced by GFP, differentiated into perineurial cells around the bundles of regenerated myelinated axons. In addition, MVSCs enhanced tight junction formation as a part of the blood-nerve barrier (BNB). Furthermore, MVSCs differentiated into perivascular cells and enhanced microvessel formation within regenerated neurovascular bundles.

**Conclusions:**

In rats with peripheral nerve injuries, the transplantation of MVSCs into the nerve conduits improved the recovery of neuromuscular function; MVSCs differentiated into perineural cells and perivascular cells and enhanced the formation of tight junctions in perineural BNB. This study demonstrates the in vivo therapeutic benefit of adult MVSCs for peripheral nerve regeneration and provides insight into the role of MVSCs in BNB regeneration.

## Background

Twenty million Americans suffer from peripheral nerve injury, which results in approximately $150 billion healthcare expenses annually in the USA [1]. Currently, the gold standard for traumatic peripheral nerve injuries is doing surgery that directly repairs the discontinuity or bridges the gap with a nerve graft or a conduit. Despite the regenerative potential of injured peripheral neurons (~ 1 mm/day) [2], the recoveries are unpredictable with unsatisfactory functional outcomes [3], especially in the conduit across critical nerve gap (larger than 10 mm). Stem cell therapy is a promising approach to promote neural tissue regeneration. To optimize in vivo the therapeutic effect, the appropriate source and differentiation stage of transplanted stem cells are crucial [4].

Several types of stem cells have been explored for peripheral nerve regeneration (PNR), including umbilical cord blood stem cells, bone marrow-derived stem cells, adipose-derived stem cells, and neural stem cells [5]. These adult stem cells have demonstrated promising therapeutic benefits with less concerns about ethics, regulation, and tumor formation, as compared to embryonic stem cells. However, the underlying mechanisms related to paracrine effect or the differentiation of transplanted cells into neurogenic or angiogenetic cells are still not well understood.

Our previous work identified a new type of adult stem cells, multipotent vascular stem cells (MVSCs) [6], from the blood vessel wall. MVSCs have a self-renewal ability and express markers of neural crest stem cells (NCSCs) such as Sox10 and Sox17. These MVSCs can be differentiated into neural cells and mesenchymal cells in vitro. In vivo investigation has shown that MVSCs can be activated by vascular injuries, become proliferative, and differentiate into smooth muscle cells (SMCs).

We postulated that MVSCs may benefit PNR by differentiating into neural and/or vascular cell types in the regenerated nerve. In this study, we transplanted MVSCs into the nerve conduits in a critical nerve gap injury model. In vivo therapeutic effects, including functional recovery and cell fate, were investigated.

## Materials and methods

### Cell isolation and characterization

MVSCs were isolated from the tunica media layers of the aorta of transgenic Sprague-Dawley (SD) rats expressing enhanced green fluorescence protein (GFP) (University of Missouri, Columbia). The cell isolation methods were described previously, using a tissue explant culture method [7]. Briefly, the tissue segments were washed three times with phosphate-buffed saline (PBS) supplemented with 1% penicillin/streptomycin (P/S). The surrounding connective tissues and adventitia were removed under a dissecting microscope. The endothelium was removed by scraping off the cell layer on the luminal surface with sterile scalpel blades. Using a tissue explant culture method, the tunica media was cut into millimeter size and placed onto the surface coated with 1% CellStart (Invitrogen) in 6-well plates. The cells were initially cultured in DMEM with 2% chick embryo extract (MP Biomedical), 1% fetal bovine serum, 1% N2 (Invitrogen), 2% B27 (Invitrogen), 100 nM retinoic acid (Sigma-Aldrich), 50 nM 2-mercaptoethanol (Sigma-Aldrich), 1% P/S, and 20 ng/ml bFGF (R&D Systems) (as phenotype maintenance medium). Cells were then seeded onto CellStart-coated dishes and maintained at 37 °C in an incubator with 5% CO_2_. Among all isolated cells, small population of cells (less than 10%) were small, round, and negative for smooth muscle myosin heavy chain (SM-MHC) expression, a mature marker of vascular SMCs [8]. These SM-MHC^−^ cells, mostly MVSCs [6], were expandable with enhanced telomerase activity as compared to SM-MHC^+^ cells.

For immunostaining, cells were fixed with 4% paraformaldehyde (PFA), permeabilized with 0.5% Triton-100 (Sigma-Aldrich), and blocked with 1% bovine serum albumin (Sigma-Aldrich). For the staining of cell markers, samples were incubated with specific primary antibodies: Sox10 (ab155279, Abcam), Sox17 (ab84990, Abcam), Ki67 (ab197234, Abcam), smooth muscle α-actin (⍺-SMA) (ab5694, Abcam), calponin-1 (CNN1) (ab233854, Abcam), CD31 (ab28364, Abcam), peripherin (ab4666, Abcam) or S100β (ab4066, Abcam) for 2 h at room temperature, washed with PBS for 3 times, and then incubated with appropriate Alexa 488- and/or Alexa 546-labeled secondary antibodies (Molecular Probes). The nuclei were stained with 4,6-diamidino-2-phenylindole (DAPI) (Invitrogen). Fluorescence images were collected using a Zeiss LSM710 confocal microscope.

### Nerve conduit fabrication

Electrospinning technique was used to produce nanofibrous nerve conduits as previously described [4]. Non-woven aligned nanofibrous nerve conduits composed of poly(l-lactide-co-caprolactone) (PLCL) (70:30, Purac Biomaterials), poly(propylene glycol) (Acros Organics), and sodium acetate (Sigma) were fabricated by using a customized electrospinning process. To make tubular scaffolds with aligned nanofibers in the longitudinal direction on the luminal surface, a rotating mandrel assembly with two electrically conductive ends and a central non-conductive section was used. The jet stream of polymer solution from the spinneret whipped between the two conductive ends, resulting in longitudinally aligned nanofibers, forming a tubular scaffold on the non-conductive portion of the mandrel. To enhance the mechanical strength of the scaffolds, the outer layers of random nanofibers were deposited onto this inner layer of longitudinally aligned fibers [9].

### Matrigel matrix formulation and preparation of tissue engineered conduits

The Matrigel we used in this study was Growth Factor Reduced (GFR) BD Matrigel Matrix (Cat. No.356230). The PLCL conduits were cut to 1.1cm-long and sterilized with ethylene oxide gas before use. MVSCs were detached and re-suspended in serum-free MVSC maintenance medium to the density of 40 million cells/ml, modified from previous protocol [10]. The cell suspension was then mixed with cold GFR matrigel solution at 1:1 ratio (volume to volume). Cells in 25 μL of matrigel was used to fill up one 1.1-cm PLCL conduit and thus the transplanted cell number was 1 million per conduit. The filled conduits were incubated in 37°C for 1 h to allow matrigel solidification. Serum-free MVSC maintenance medium was then added to cover the tissue-engineered nerve conduits for overnight culture in 37°C incubator before surgery.

### In vivo transplantation of stem cells and nerve conduits

To investigate the in vivo therapeutic effect, 18 adult SD rats were randomly divided into three treatment groups (acellular, MVSC, and autograft, *N* = 6 in each group). MVSC transplantation was performed using allogenic cells which were isolated from GFP rats. All animal experimental procedures were approved by the Animal Care and Use Committee at UC Berkeley and were carried out according to the institutional guidelines. Adult female SD rats (Charles River) weighing 200–250 g were used in all treatment groups. For nerve conduit implantation, an incision was made over the skin above the hip joint with a sterile scalpel. Under a surgical microscope, the sciatic nerve was isolated and severed with a microscissor at two spots to make a 10-mm gap. Then, the tissue-engineered nerve conduits (11 mm in length, 1.5 mm in inner diameter), with or without MVSCs, were inserted between the two nerve stumps and then sutured with 9-0 nylon monofilament sutures (Ethilon, Ethicon). The overlying muscle layers and skin were sutured with 4-0 absorbable sutures (polydioxanone, Ethicon) to close the surgery site. For the autograft group, 10-mm segment of sciatic nerve was transected and microscopically repaired in a reverse fashion. After 1-month transplantation, nerve regeneration was assessed by electrophysiology. To confirm the histologic evidence, all animals were euthanized and the conduits were harvested, fixed in 4% PFA for immunohistochemistry (IHC) analysis.

### Electrophysiology

Electrophysiology testing was performed to evaluate the functional recovery of regenerated nerve by following the previous method [4]. In brief, the rat sciatic nerve was exposed, and electrical stimuli (single-pulse shocks, 1 mA, 0.1 ms) were applied to the native sciatic nerve trunk at the point 5 mm proximal to the graft suturing point. Amplitude of the depolarization was recorded as compound muscle action potential (CMAP) on the gastrocnemius belly from 1 to 12 V or until a supramaximal CMAP was reached. Normal CMAP from the unoperated contralateral side of the sciatic nerve was also recorded for comparison, at the same level of the sciatic nerve. Grass Tech S88X Stimulator (Astro-Med Inc.) was used for the test, and PolyVIWE16 data acquisition software (Astro-Med, Inc.) was used for recording. Recovery rate is the ratio of injured hindlimb’s CMAP to contralateral normal hindlimb’s CMAP of a rat [4]. After electrophysiological evaluation, the rats were sacrificed to harvest the regenerated nerve for histological examination.

### IHC analysis

The nerve conduits were harvested and fixed in 4% PFA at 4 °C for 2 h. After being washed with PBS, the tissues were cryoprotected with 30% sucrose in PBS at 4 °C overnight, and were then embedded in optimum cutting temperature compound, followed by being frozen at − 80 °C. The frozen samples were cryosectioned longitudinally and transversely at − 20 °C for the thickness of 10 μm. The slices were placed onto Superfrost plus slides and stored at − 20 °C. IHC staining was performed for histological analysis. The slices were permeabilized with 0.5% Triton X-100 in PBS for 30 min, blocked with 4% normal goat serum in PBS for 1 h, and then incubated overnight at 4 °C with primary antibodies. The slides were then washed with PBS and incubated with secondary antibodies for 1 h at room temperature. After further PBS washing, the coverslips were mounted and viewed with a fluorescent microscope (Zeiss). The primary antibodies used for IHC analysis in this study were as follows: neural filament-medium polypeptide (NFM) (ab7794, Abcam), S100β (ab4066, Abcam), Claudin-1 (ab15098, Abcam), CD31 (ab28364, Abcam), ⍺-SMA (ab5694, Abcam), and CNN1 (ab233854, Abcam). Fluorescence-tagged anti-mouse and anti-rabbit secondary antibodies (Abcam) were used.

### Statistical analysis

The data was reported as mean ± standard error of mean, unless otherwise described. Comparisons among values for groups greater than two were performed by one-way analysis of variance, and differences between the groups were then determined using a Tukey’s post hoc test. For all experiments, a value of *p* < 0.05 was considered statistically significant. GraphPad Prism software (version 8.0) was used for all statistical analyses.

## Results

### Characterization of MVSCs isolated from rat aorta

**Fig. 1:**
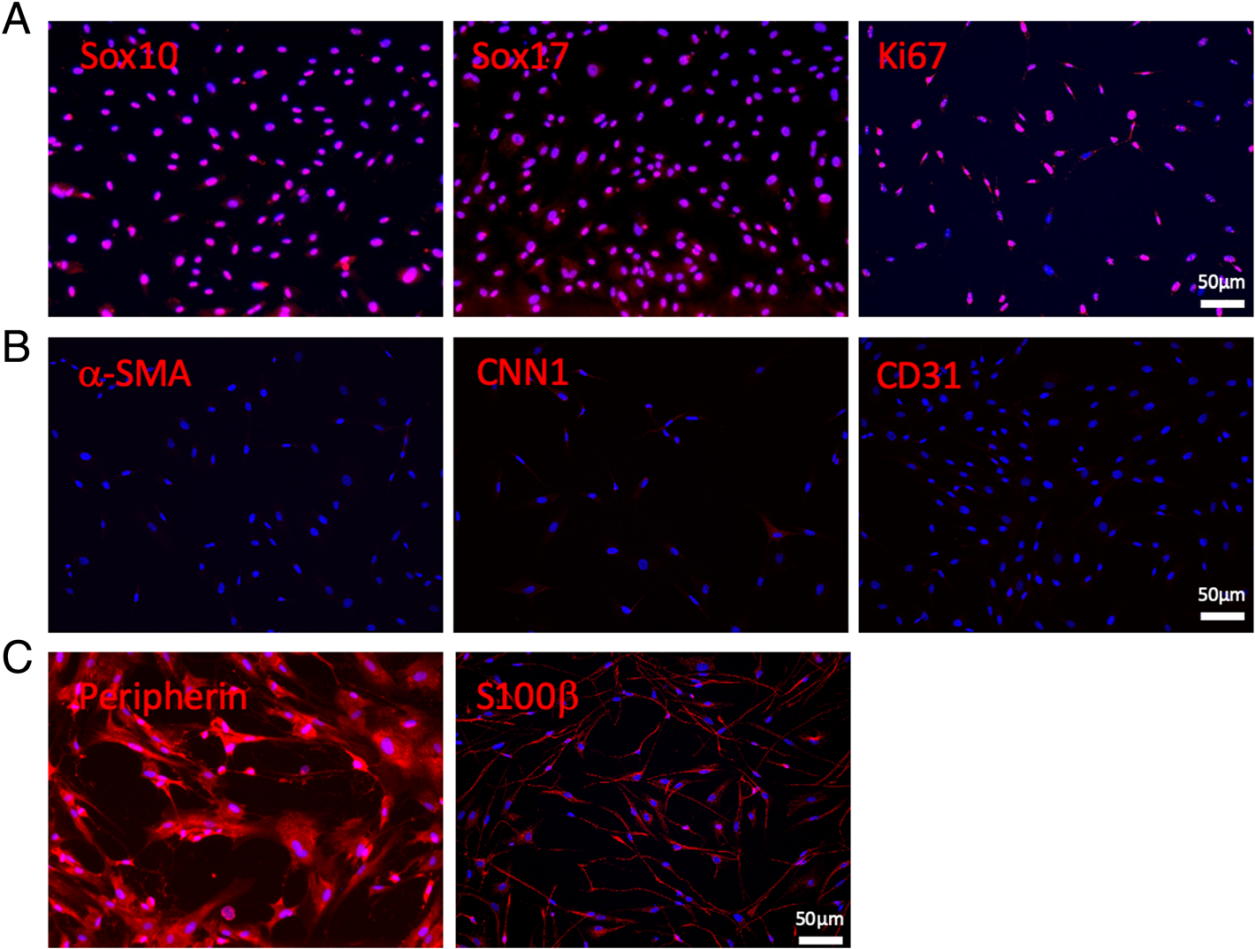
In vitro characterization and differentiation of MVSCs isolated from rat aorta. **a** MVSCs were isolated from the aorta of GFP rats by using a tissue explant culture method, expanded, and stained for Sox10, Sox17, and Ki67. **b** MVSCs were stained for vascular cell markers α-SMA, CNN1, and CD31. **c** MVSCs were differentiated into peripheral neurons (Peripherin^+^) and Schwann cells (S100β^+^) in vitro. Red color indicates positive staining for individual marker; blue color indicates positive DAPI staining. Scale bar, 50 μm

In vitro characterization of SM-MHC^−^ cells revealed positive expression of stem cell markers Sox10 and Sox17, and these cells were highly proliferative (Ki67 staining) and expandable (Fig. 1a). MVSCs were negative for ⍺-SMA and CNN1 and distinguishable from vascular SMCs; in addition, MVSCs were negative in CD31 expression, indicating a non-endothelial origin (Fig. 1b). After an in vitro induction, MVSCs differentiated into the neural lineage, such as peripheral neuron (peripherin^+^) and Schwann cell (S100β^+^) (Fig. 1c).

### MVSCs promoted in vivo functional recovery in critical nerve gap injuries

**Fig. 2:**
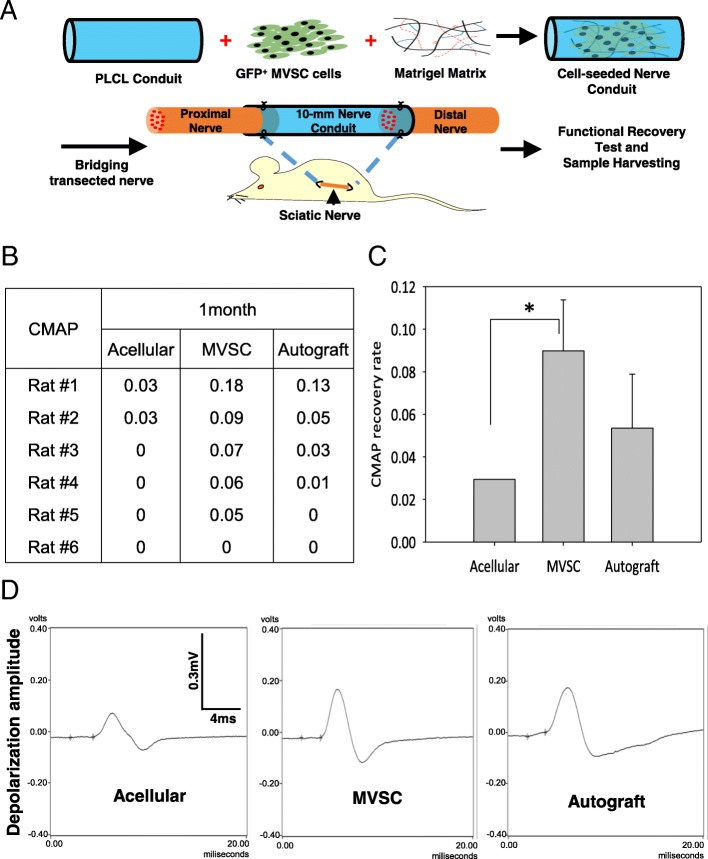
In vivo evaluation of functional recovery. **a** Schematic showing that GFP^+^ MVSCs (allograft) were mixed with Matrigel matrix and injected into a PLCL nerve conduit that was used to bridge a critical gap of injured nerve in Sprague-Dawley rats. **b** Recordable amplitude of electrophysiological depolarization in each rat following 1-month recovery. **c** CMAP of the sciatic nerve was measured following 1-month recovery. Recovery rate is the ratio of injured hindlimb’s CMAP to contralateral normal hindlimb’s CMAP of a rat. For CMAP recovery, the acellular group and MVSC-engrafted group showed a significant difference (*p < 0.05). Bars represent mean ± standard error. **d** Representative curves of CMAP (rat #1 of each group) are shown for the acellular group, the MVSC-engrafted group, and the autograft group

Adult SD rats were randomly divided into three treatment groups (acellular, MVSC, and autograft), in order to compare the therapeutic benefits. HA-collagen gel with MMP-targeting crosslinker was used for cell delivery into the nerve conduits. Hydrogel only (acellular) or MVSCs in hydrogel were injected into 10-mm PLCL nanofibrous electrospun conduit to bridge the critical sciatic nerve gap in the left hindlimb (Fig. 2a). For the autograft group, the 10-mm transected sciatic nerve was reversed (proximal vs. distal) and sutured back in situ. The functional recovery of the regenerated nerve was evaluated by electrophysiology testing. CMAP of the injured sciatic nerve and the contralateral intact nerve were measured and compared. One month after surgery, the MVSCs and autograft groups consistently showed a higher rate of detectable CMAP (Fig. 2b, c). For the rats with detectable CMAP, the recovery rate of each rat was calculated as a percentage of CMAP over the uninjured contralateral sciatic nerve. A significant difference was observed between the MVSC group and the acellular group (*p* < 0.01) but not between the MVSC group and the autograft group (Fig. 2d). The latency period of recordable depolarization did not show a significant difference among the three groups (data not shown).

### MVSCs formed perineural structure and tight junctions in the regenerated nerve

**Fig. 3:**
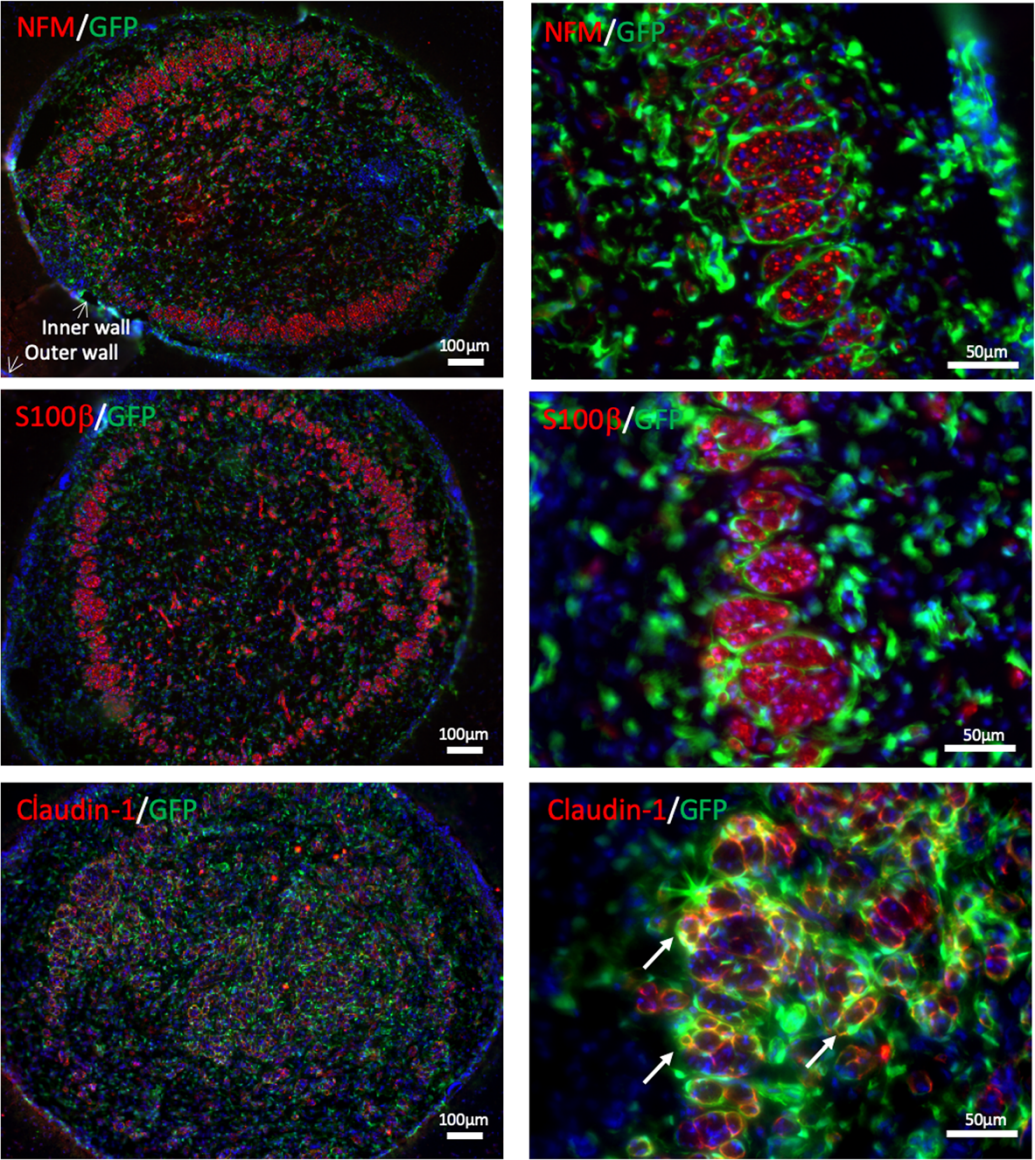
MVSCs formed perineurium-like structure in the regenerated nerve. IHC analysis of cross-sections in the middle of the nerve conduits was performed after 2-month transplantation. The samples were stained for NFM (axons), S100β (Schwann cell), and Claudin-1 (tight junction). Red color indicates positive staining for individual marker; blue color indicates positive DAPI staining; green color indicates GFP^+^ MVSC-derived cells. Right column: magnified images (scale bars: 100 μm in the left column, 50 μm in the right column)

Two month after nerve injury, the regenerated nerve across the conduit was harvested for histological evaluation. IHC staining revealed positive staining of NFM (axon) and S100β (Schwann cells) across the cross-section area, with more axons and Schwann cells near the conduit wall (Fig. 3), suggesting that myelinated axons regenerated more effectively near the nerve conduit surface. Higher resolution analysis demonstrated that GFP^+^ MVSCs formed a perineurium-like structure around bundles of axons (Fig. 3). In addition, IHC staining showed co-localization of GFP with Claudin-1 (Fig. 3), an important tight junction protein and a perineurium marker [11, 12]. This result suggested that the transplanted MVSCs could facilitate the formation of tight junctions in the blood-nerve barrier (BNB) within the regenerating nerve.

**Fig. 4:**
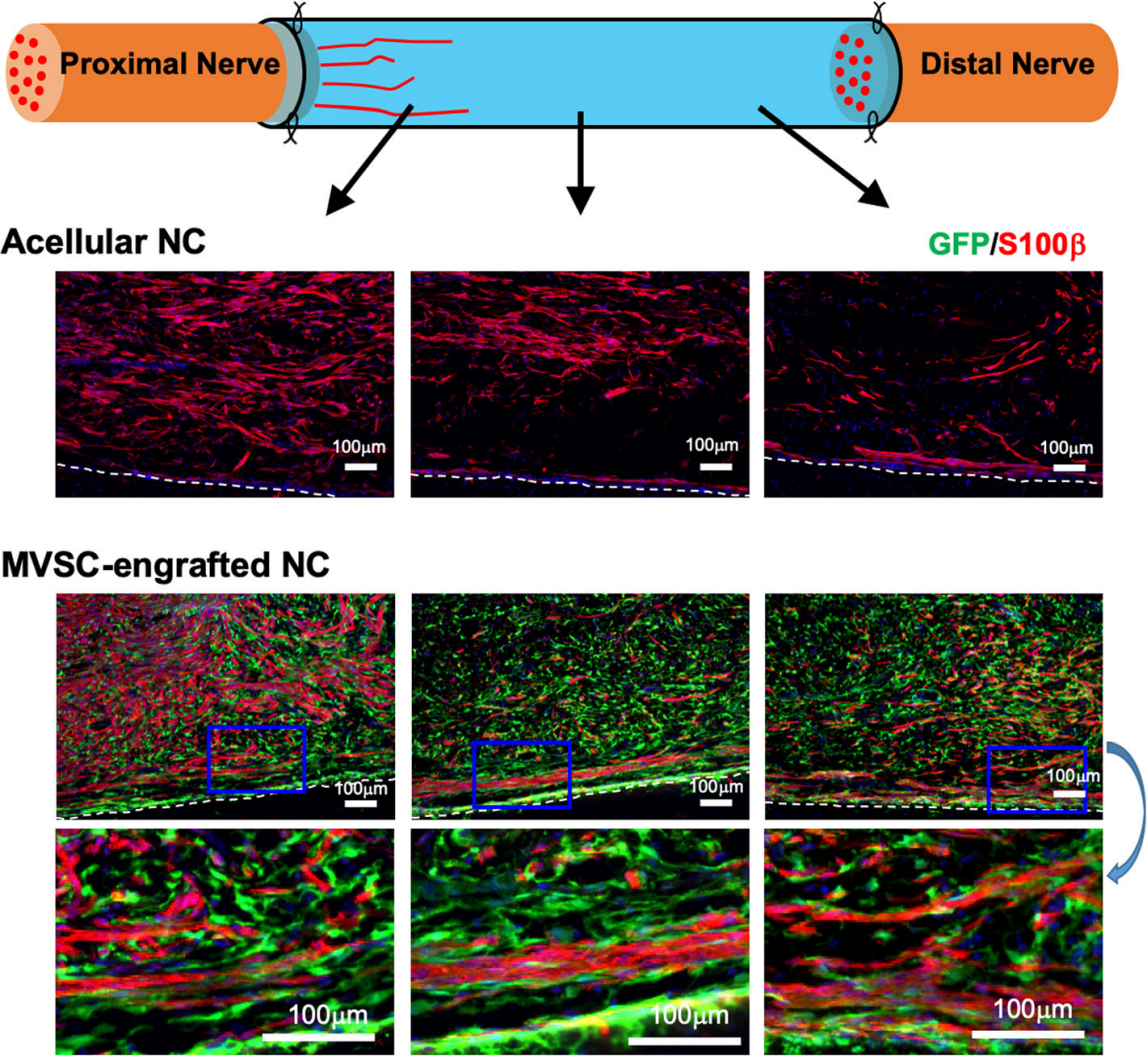
Longitudinal analysis of the regenerated nerve after 1-month transplantation. Longitudinal sections showed the distribution of Schwann cells (S100β^+^) and GFP^+^ MVSCs in the proximal, middle, and distal segments of the nerve conduits. Red color indicates positive staining for S100β; blue color indicates positive DAPI staining; green color indicates GFP^+^ cells. Scale bar, 100 μm

Longitudinal sections of the regenerated nerve demonstrated that disordered Schwann cell alignment was observed in acellular nerve conduit, with diminished myelination (S100β) at distal regenerated nerve (Fig. 4). In contrast, the MVSC-transplanted group demonstrated well-aligned S100β^+^ cells with GFP^+^ staining in the entire length of the conduit, implying that MVSCs could facilitate the recruitment of Schwann cells and play a supporting role in axon growth.

### MVSCs enhanced angiogenesis and differentiated into perivascular cells within regenerated nerve

**Fig. 5:**
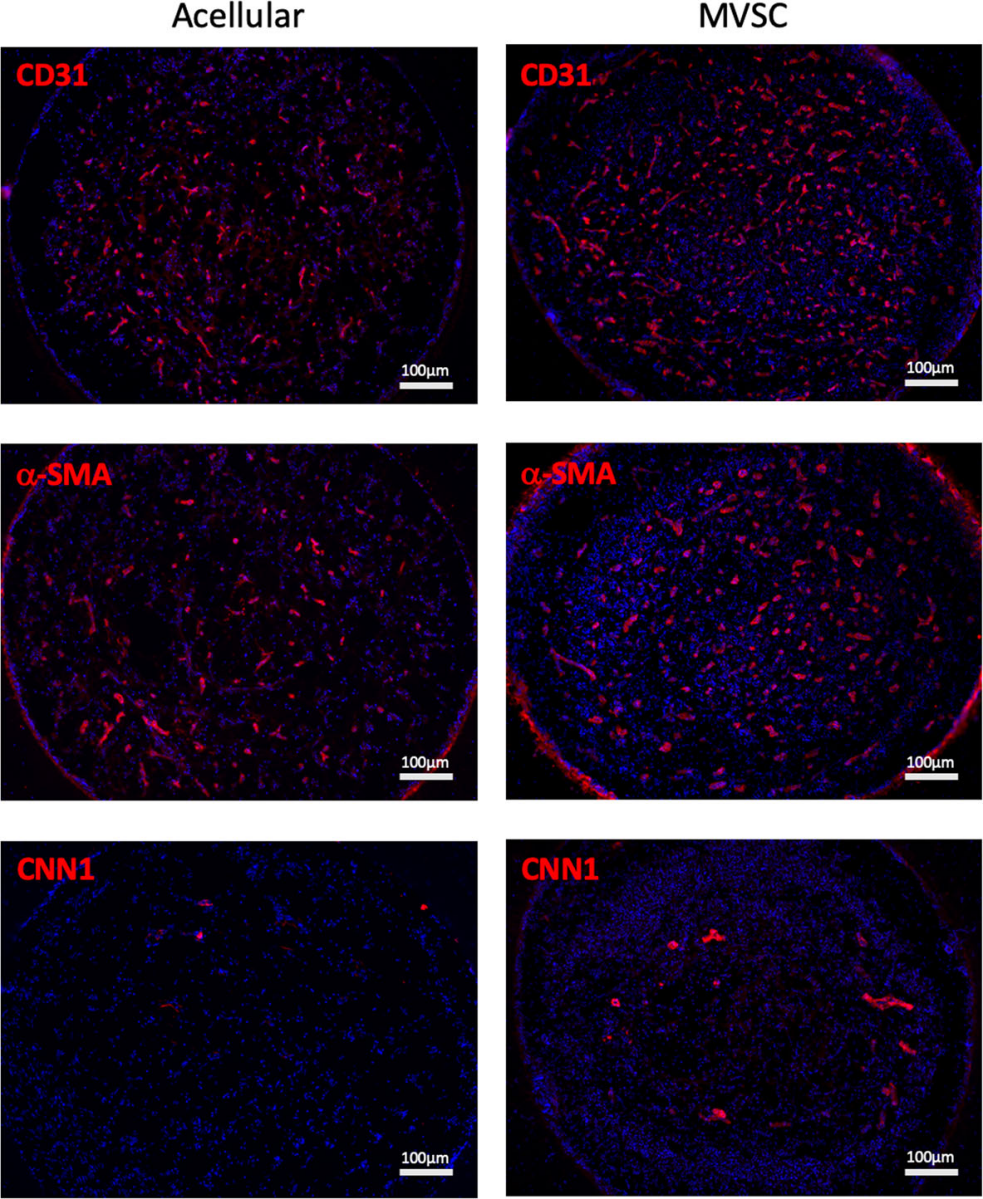
Microvessel formation in the regenerated nerve. IHC staining of CD31, α-SMA, and CNN1 for the comparison of microvessel formation at the middle segments of the regenerated nerve in acellular conduits and MVSC-transplanted conduits at 2-month post-transplantation. Red color indicates positive staining for individual marker; blue color indicates positive DAPI staining. Scale bars, 100 μm

**Fig. 6:**
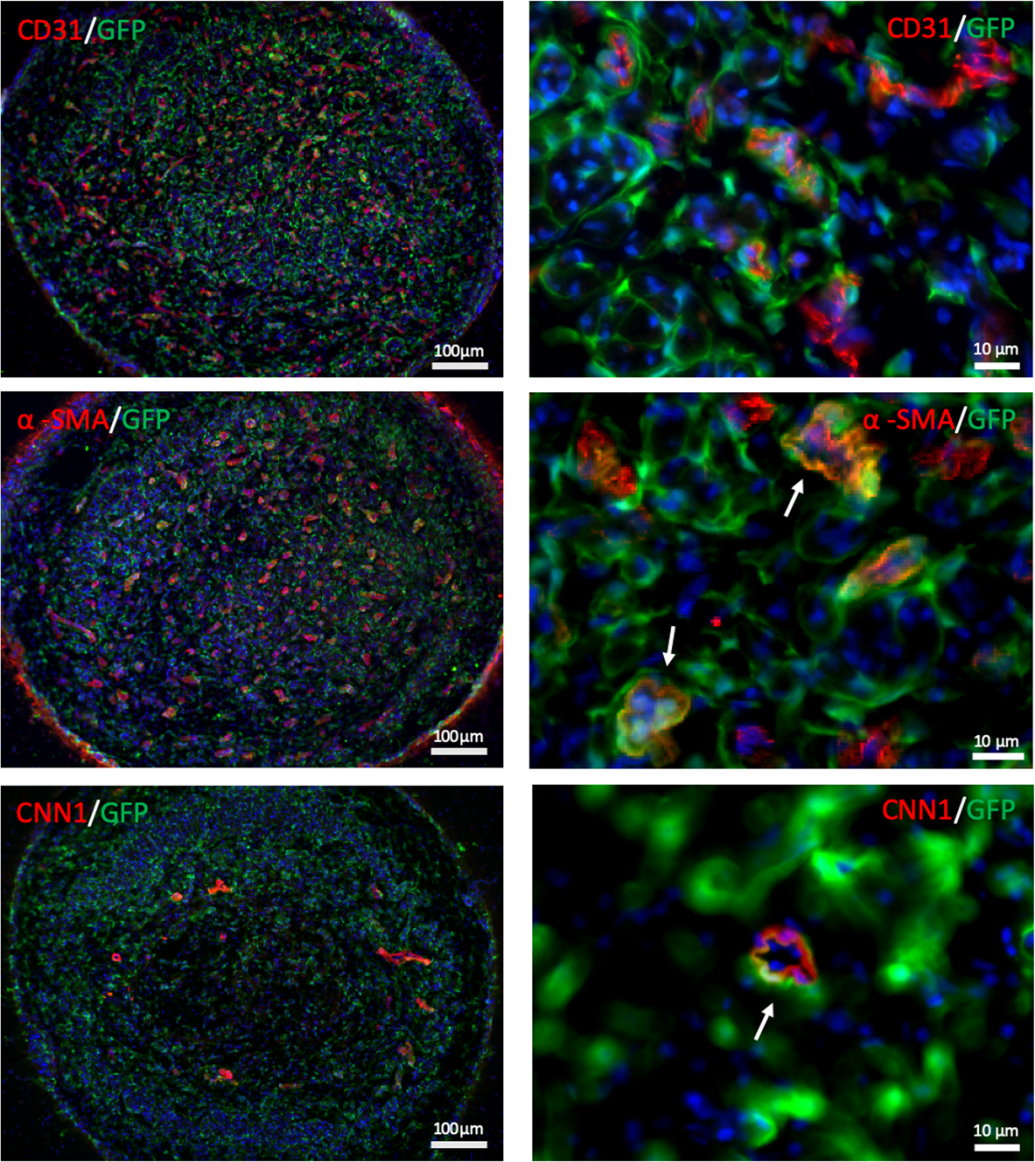
MVSCs differentiated into perivascular cells in regenerating nerve tissue. IHC analysis of the middle segment of the MVSC-transplanted conduits at 2-month post-transplantation was performed by staining for endothelial cell marker (CD31) and perivascular cell markers (α-SMA and CNN1). MVSCs were labeled by GFP. Red color indicates positive staining for individual marker; blue color indicates positive DAPI staining; green color indicates GFP^+^ cells (scale bars: 100 μm in the left column, 10 μm in the right column)

To further investigate the functionality of transplanted MVSCs, we performed IHC analysis of vascular markers. CD31 (endothelial marker) staining showed more microvessels in the MVSC-transplanted group (Fig. 5). In addition, the number of ⍺-SMA^+^ and CNN1^+^ cells also significantly increased in the MVSC-treated group. Close examination of the regenerated nerve in the MVSC-transplanted group demonstrated CD31^+^ tubular structure throughout the cross-section of the regenerated nerve, which was surrounded by GFP^+^ MVSCs (Fig. 6). Co-localization of GFP^+^ MVSCs and ⍺-SMA^+^ tubular structure was observed, indicating that the MVSCs differentiated into perivascular cells of microvessels. In addition, CNN1 is an intermediate marker of SMCs and a regulatory protein involved in SMC contractility [13, 14]. Co-localization of GFP^+^/CNN1^+^ signal further implied that MVSC could differentiate into perivascular cells to stabilize the microvessels in the regenerated nerve.

## Discussion

Regeneration of the peripheral nerve across a critical gap remains a great challenge and unsolved problem for decades. Appropriate stem cell therapy still holds the holy grail to promote regeneration by differentiating into neuron and/or glial cells or supporting endogenous neurogenesis with paracrine effect. Pericytes are believed to function as progenitor cells with great potential for regenerative medicine [15], including neural cell differentiation and regeneration [16, 17]. However, the current view toward endogenous pericytes as multipotent tissue-resident progenitors is still under debate [18]. MVSCs express NCSC markers and can be expanded rapidly in culture and differentiated into neural lineage cells along with SMCs, which clearly distinguishes MVSCs from other known progenitor cells, making it unique for the regeneration of neurovascular structures. In this study, the neurogenic potential of MVSCs was verified by the expression of NCSC markers (Sox10^+^, Sox17^+^) and the differentiation into glial cells (S100β^+^) and peripheral neurons (peripherin^+^). Implantation of MVSCs into critical nerve gap enhanced the functional recovery, demonstrating in vivo therapeutic potential.

Microvessels in the peripheral nerve are critical to support cell survival and regeneration. In this study, transplanted MVSCs enhanced newly formed microvessels in the regenerated nerve, as compared to the acellular group. Cell fate analysis further revealed that the transplanted MVSCs differentiated into perivascular cells (⍺-SMA^+^/CNN1^+^). Hence, these transplanted MVSCs may support and stabilize the newly formed microvessels.

Regenerated microvessels across the nerve gap can act as a scaffold to guide Schwann cells for efficient adhesion and migration, leading to enhanced axon growth and myelination [19]. Interestingly, MVSC transplantation increased the recruitment of Schwann cells, showing striking difference near the distal end of the nerve conduits. It is possible that MVSCs enhance microvessel formation, which in turn facilitates the directional migration of Schwann cells toward the distal end. Alternatively, MVSCs may help recruit Schwann cells via paracrine signaling to enhance axon myelination. Although MVSCs have potential to differentiate into Schwann cells or peripheral neurons in vitro, we did not detect MVSC differentiation into neural lineages in vivo, suggesting that MVSCs tend to differentiate into mesenchymal lineages in the regenerating nerve. These findings underscore the difference between adult MVSCs and NCSCs derived from induced pluripotent stem cells. As shown in our previous work, NCSCs in nerve conduits differentiated into Schwann cells to enhance the myelination of axons [9].

The transplanted MVSCs in the nerve conduits formed a perineural structure, wrapping around the regenerated axon bundles. We further demonstrated that transplanted MVSCs co-localized with claudin-1^+^ cells, suggesting the formation of tight junction in BNB [20]. BNB protects peripheral nerves from immunologic attacks and helps to maintain the stability of the endoneurial-endothelial interface [21]. As far as we know, this is the first demonstration that the transplantation of adult stem cells facilitates BNB formation in PNR. This effect may be attributed to the differentiation of MVSCs into perivascular cells to stabilize the microvessels, or to the formation of a perineurium-like structure by MVSCs, which warrants further investigation.

## Conclusions

In summary, this study demonstrates the novel functions of transplanted MVSCs in promoting PNR, including Schwann cell recruitment, differentiation into perineural and perivascular cells, and tight junction formation in BNB. These findings provide insight into the interactions of neural cells and vascular cells in regenerating neurovascular bundles and give rise to new strategies for nerve regeneration.

## Data Availability

All data generated or analyzed during this study are included in this published article and also available from the corresponding author on reasonable request.

## Abbreviations

⍺-SMA: Smooth muscle α-actin
BNB: Blood-nerve barrier
CMAP: Compound muscle action potential
CNN1: Calponin-1
DAPI: 4,6-Diamidino-2-phenylindole
GFP: Green fluorescent protein
GFR: Growth factor reduced
IHC: Immunohistochemistry
MVSCs: Multipotent vascular stem cells
NCSCs: Neural crest stem cells
NFM: Neural filament-medium polypeptide
P/S: Penicillin/streptomycin
PBS: Phosphate-buffed saline
PFA: Paraformaldehyde
PLCL: Poly(l-lactide-co-caprolactone)
PNR: Peripheral nerve regeneration
SD: Sprague-Dawley
SMCs: Smooth muscle cells
SM-MHC: Smooth muscle myosin heavy chain
